# Thermophysical Properties of Nanocolloids and Their Potential Applications

**DOI:** 10.3390/nano13010217

**Published:** 2023-01-03

**Authors:** Alina Adriana Minea

**Affiliations:** Materials Science and Engineering Faculty, “Gheorghe Asachi” Technical University of Iasi, 700050 Iasi, Romania; aminea@tuiasi.ro

This Special Issue is a continuation of the previous successful Special Issue, entitled “Future and Prospects in Nanofluids Research”, co-edited by the present Editor and dedicated to the topic of “Thermophysical Properties of Nanocolloids and Their Potential Applications”.

Nanocolloids are not a new concept. They include popular new fluids, which are identified in the literature as nanofluids. The term “nanofluid” is applied to a variety of these base fluids that are enhanced with nanoparticles. Nevertheless, a term that is wider in scope is “nanocolloids”. This Special Issue contains original high-quality research papers covering the most recent advances in research on nanocolloid thermophysical properties, as well as comprehensive reviews addressing the relevant state-of-the-art topics in the field of nanoparticle suspensions applied in fluids, together with their relevant practical applications. A novelty of this Special Issue is that it was designed to attract papers that can be viewed as opinions and open questions, as well as critical assessments of this particular topic. Papers addressing areas of research beyond engineering were also encouraged in order to broaden the application potential of nanocolloids and to facilitate the formation of new opinions.

This Special Issue covers the characterization of new nanoparticle-enhanced fluids/nanocolloids, focusing on the relevant and innovative applications of such an approach. These base fluids can be water, glycols, oils, molten salts, or ionic liquids, with the research topics covering but not limited to most of the applications related to heat transfer, lubrication, and chemical engineering. Applications involving innovative systems are also explored.

We gratefully acknowledge the authors and reviewers who participated in the creation of this Special Issue and, thus, their contribution to the development of nanocolloid research.

Several articles were received, and with the help of our experienced reviewers, 10 papers were selected for publication [1,2,3,4,5,6,7,8,9,10]. From a statistical data perspective, the reader should note that the articles had already received 29 citations and 9243 visualizations at the time of the writing of this Editorial. Of the 10 published articles, 2 are reviews dedicated to the topics of “Advances and Challenges in Measuring the Thermal Conductivity of Nanofluids” [9] and “Ionic Liquids-Based Nanocolloids—A Review of Progress and Prospects in Convective Heat Transfer Applications” [2].

Essentially, all the papers were written by recognized experts in the field of new fluid development. The Special Issue Editor thanks the authors for choosing to publish their findings in this very important research area in the *Nanomaterials* journal.

The first article originates from University of Vigo and Institut für Luft-und Kältetechnik Dresden labs and is an experimental analysis of the heat transfer of nanofluids based on graphene nanoplatelets diluted in water [1]. The authors used an experimental facility to evaluate the heat transfer performance based on a turbulent regime, obtaining an enhancement of the nanofluid of up to 13%. Another experimental study was conducted in regard to the thermophysical properties of ammonia and carbon nanomaterials (graphene and single-wall carbon nanotubes) dispersed in [BMIM] BF4 ionic liquid [3]. The authors’ results indicate that by adding a small amount of nanomaterial to the ionic liquid, the nanofluid thermal conductivity can be improved. Other papers addressed different types of nanofluids, such as zinc oxide [8] or graphene [4], and base fluids, such as PEG 400 [8] or ionic liquids [5]. An interesting paper explored the results of an experimental and theoretical study of the two-photon-assisted SRMS in Ag and ZnO nanocolloids in the nanosecond-to-picosecond pulse width domain [10].

Considering the high quality of the published articles, as well as their research impact, the Editor decided to publish a second part of this Special Issue, which was recently launched, entitled “Thermophysical Properties of Nanocolloids and Their Potential Applications Ⅱ” (see: https://www.mdpi.com/journal/nanomaterials/special_issues/8JT5M5A61Y).

## Data Availability

No data were used for this article.

## References

[1] Calviño U., Vallejo J., Buschmann M., Fernández-Seara J., Lugo L. (2021). Analysis of Heat Transfer Characteristics of a GnP Aqueous Nanofluid through a Double-Tube Heat Exchanger. Nanomaterials.

[2] Minea A., Sohel Murshed S. (2021). Ionic Liquids-Based Nanocolloids—A Review of Progress and Prospects in Convective Heat Transfer Applications. Nanomaterials.

[3] Huminic G., Huminic A. (2021). Thermophysical Properties of NH3/IL+ Carbon Nanomaterial Solutions. Nanomaterials.

[4] Ali N. (2022). Graphene-Based Nanofluids: Production Parameter Effects on Thermophysical Properties and Dispersion Stability. Nanomaterials.

[5] Svobodova-Sedlackova A., Huete-Hernández S., Calderón A., Barreneche C., Gamallo P., Fernandez A. (2022). Effect of Nanoparticles on the Thermal Stability and Reaction Kinetics in Ionic Nanofluids. Nanomaterials.

[6] Han Y., Yang Y., Mallick T., Wen C. (2022). Nanoparticles to Enhance Melting Performance of Phase Change Materials for Thermal Energy Storage. Nanomaterials.

[7] Bohus M., Ba T., Hernadi K., Gróf G., Kónya Z., Erdélyi Z., Parditka B., Igricz T., Szilágyi I. (2022). Thermal Conductivity Enhancement of Atomic Layer Deposition Surface-Modified Carbon Nanosphere and Carbon Nanopowder Nanofluids. Nanomaterials.

[8] Minea A., El-Maghlany W., Massoud E. (2022). Heat Transfer Analysis of Nanocolloids Based on Zinc Oxide Nanoparticles Dispersed in PEG 400. Nanomaterials.

[9] Souza R., Faustino V., Gonçalves I., Moita A., Bañobre-López M., Lima R. (2022). A Review of the Advances and Challenges in Measuring the Thermal Conductivity of Nanofluids. Nanomaterials.

[10] Erokhin A., Bulychev N., Parkevich E., Medvedev M., Smetanin I. (2022). Stimulated Thermal Scattering in Two-Photon Absorbing Nanocolloids under Laser Radiation of Nanosecond-to-Picosecond Pulse Widths. Nanomaterials.

